# Fiber Optic Refractive Index Sensors Based on a Ball Resonator and Optical Backscatter Interrogation

**DOI:** 10.3390/s20216199

**Published:** 2020-10-30

**Authors:** Madina Shaimerdenova, Takhmina Ayupova, Marzhan Sypabekova, Daniele Tosi

**Affiliations:** 1 School of Engineering and Digital Sciences, Nazarbayev University, Nur-Sultan 010000, Kazakhstan; madina.shaimerdenova@nu.edu.kz (M.S.); takhmina.ayupova@nu.edu.kz (T.A.); 2 Laboratory of Biosensors and Bioinstruments, National Laboratory Astana, Nur-Sultan 010000, Kazakhstan; msypabekova@nu.edu.kz; 3 School of Medicine, Nazarbayev University, Nur-Sultan 010000, Kazakhstan

**Keywords:** optical fiber sensors, optical resonators, refractive index sensors, optical backscatter reflectometry, ball resonator

## Abstract

In this work, we introduced fabrication and interrogation of simple and highly sensitive fiber-optic refractive index (RI) sensors based on ball resonators built on the tip of single-mode fibers. The probes have been fabricated through a CO_2_ fiber splicer, with a fast (~600 s) and repeatable method. The ball resonator acted as a weak interferometer with a return loss below −50 dB and was interrogated with an optical backscatter reflectometer measuring the reflection spectrum. The ball resonators behaved as weak interferometers with a shallow fringe and a spectrum that appeared close to a random signal, and RI sensitivity could be measured either through wavelength shift or amplitude change. In this work, we reported four samples having sensitivity ranges 48.9–403.3 nm/RIU and 256.0–566.2 dB/RIU (RIU = refractive index unit). Ball resonators appeared as a sensitive and robust platform for RI sensing in liquid and can be further functionalized for biosensing.

## 1. Introduction

Optical fiber refractive index (RI) sensors are an emerging technology with significant potential for biomedical applications [1]. RI sensors represent the platform for fiber-optic biosensors, which functionalize the design of RI sensing to selective detection of a biological analyte [2]. Optical fiber biosensors have been demonstrated for the detection of proteins, tumor biomarkers, antibodies, and small cells, among others [3,4,5,6].

From a technological standpoint, RI sensors and biosensors based on fiber-optic systems have been reported using different technologies. Plasmonic devices have been reported in several configurations using a metallic thin film at the interface between the glass fiber and the outer analyte to host surface plasmon resonance (SPR) [7]. SPR devices have high sensitivity, up to several thousand nm/RIU (refractive index units), and a simple design [8]; however, they usually use a multimode fiber, require a polarization control system, as SPR resonance is polarization-dependent, and operate in transmission, which complicates the design of a reflective probe. Spectrometers used to detect wide-band SPR sensors have coarse resolution [9].

Gratings and interferometers have been investigated significantly and represent an alternative to SPR, with the significant difference of operating with single-mode fibers (SMFs) and in the infrared range, typically in the third optical window around 1550 nm.

Fiber Bragg grating (FBG) RI sensors have been proposed, manipulating the structure of a standard grating to sense the environment around the fiber; tilted FBG, etched FBG, etched-tilted FBG, and plasmonic FBG devices have been proposed, achieving a sensitivity of several tens of nm/RIU, using FBG interrogators that have picometer-level wavelength resolution [10,11,12,13].

Compact interferometers have also been proposed for RI sensing, designing structures that convert RI changes into a variation of the optical path difference of a microcavity [14]. Solutions based on Fabry–Perot interferometry, single-multi-single mode interferometers, polarization-based Sagnac interferometers, and tapers have been reported, with sensitivity ratings similar to SPR sensors, and interrogated through optical spectrum analyzers [15,16,17,18].

The main drawback of gratings and interferometers for RI sensing and biosensing is the poor scalability in terms of fabrication throughput and potential costs. While the fabrication of gratings is a consolidated process, it is relatively cumbersome and slow, and requires several steps; additional manufacturing steps, such as wet-etching, which makes the fiber brittle, or coating the fiber end to obtain the transmission-reflection spectrum, contribute to increasing the complexity [19]. Similar considerations can be drawn for interferometers, which require multiple splicing steps or fiber tapering. In general, interferometers have low fabrication yield, particularly for microfibers or micrometer-thick tapers with poor mechanical robustness, which can also compromise the functionalization steps to covert the RI sensor into a selective biosensor.

In this work, we proposed a ball resonator structure for RI sensing, solving many of the problems that gratings and interferometers present in terms of fabrication ease and robustness. The ball resonator behaved as a weak multipath interferometer, with a weak reflection spectrum detected on the same input fiber.

Ball resonators, also called microsphere resonators, appear to be one of the most significant types of whispering gallery mode (WGM) resonators. The main advantages of microsphere resonators are that they can be easily manufactured from materials of different natures, are robust, and have a high-quality factor [20]. There are a few ball resonators fabrication methods, such as fusion splicing, CO_2_ splicing, and the melting process. During fusion splicing, an electric arc was used to fabricate a microsphere on the tip of an uncoated cleaved optical fiber, which led to the creation of two microspheres of 250 and 255 µm [21]. Another group also produced a resonator from a short piece of fiber (around 4 cm) with a fusion splicer [22]. In their study, a series of electric arc charges progressively allowed the optical fiber tip to melt in order to produce a microsphere. The diameter of a ball resonator depended on the number of discharges, where a high number of charges produced a larger diameter of the ball resonator. Subsequently, the fusion splicer was able to obtain a microsphere of a diameter of 171 μm.

It is of great importance that resonators have been exploited in numerous sensing applications. As mentioned before, due to changes in the outer refractive index of the surface of the microsphere, amplitude change and frequency shift can be observed, leading to the fact that the device’s sensitivity is existent. In the early 2000 s, a research group studied the sensing of bovine serum albumin in a phosphate buffer with a microsphere device [23]. They identified this system, consisting of a microsphere structure coupled to a tapered fiber, as a microsphere resonator. Later, in 2008, the same research group also found that greater sensitivity of the resonator can be achieved by decreasing ball diameter.

**Figure 1:**
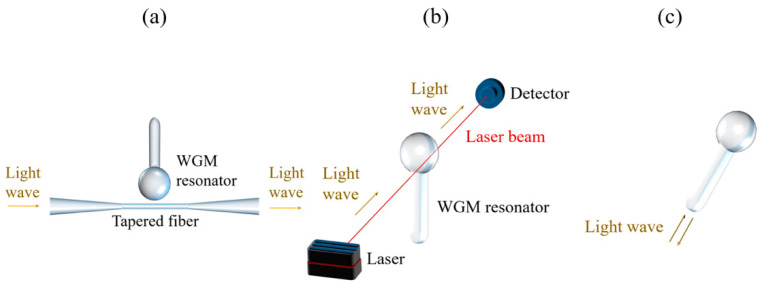
Schematics of the signal detection system of whispering gallery mode (WGM) microresonator. (**a**) Sketch of an optical WGM microresonator coupled to a tapered fiber [24], where the input light was guided inside the tapered fiber from one end, and the resulting signal was detected at another end. (**b**) WGM biosensor platform, where input optics guided the light beam through the resonator, which was then detected at output optics [25]. (**c**) The detection system presented in this work was based on input light being guided inside the ball resonator, and the resulting back-reflected output signal was detected.

In the proposed work, we presented fiber ball resonators for RI sensing, with diameters around 500 µm, fabricated on an SMF fiber. Due to low reflectivity, the spectra of ball resonators were detected with an optical backscatter reflectometer (OBR), suited for detecting weak spectra with picometer-scale resolution; the schematics of the detection system presented in this work vs. the work of our colleagues is shown in Figure 1.

The reflection spectrum of the ball resonators appeared similar to a random signal due to the weak fringes of the interferometric structure; spectra have a sensitivity that can be measured either as a wavelength shift or as an intensity change. In the following, we will discuss the fabrication and interrogation of ball resonators, discussing the results on different samples of sensors.

## 2. Fabrication and Interrogation

Ball resonators have been used for light coupling and biosensing, using several types of excitation methods. The key working principle is to convert the spherical shape of the ball structure into a resonator capable of having interference fringes. One of the most popular configurations is proposed by Soria et al. [26]; the light enters the resonator at about 30° angle, forming a hexagonal light path with resonance wavelength that is a multiple of the radius of the resonator. This configuration is also used in conjunction with fiber tapers, as in Figure 1a. Alternatively, fluidic systems for biosensing have been designed with the method of Ilchenko et al. [27], where light enters the resonator at a larger angle, and the Fresnel reflection on the borders of the ball resonator provides the wavelength-dependent pattern. These methods lead to high-Q resonators, characterized by wide fringe visibility and free spectral range controlled by the radius of the ball. The drawback is that such a wide angle of excitation cannot be generated with an SMF fiber due to its low numerical aperture; hence, external excitation systems, such as in Figure 1a,b, have been used.

In this work, the same fiber terminated by the ball resonator did both the light delivery and RI sensing tasks. In this case, the reflection spectra were similar to those observed by Watkins et al. [28] and looked similar to random signals due to the weak and chaotic interferometric structure of the ball. Compared to the method of Watkins et al., the resonators proposed here had a more spherical shape and a much faster, simpler, and more repeatable method of fabrication. However, they had lower reflectivity, which required a very sensitive detector, such as the OBR, for detection. As in Watkins’s and similar works [29], the spectrum of the resonator did not appear as a high-fringe regular interferometer, but rather it displayed several low fringes spread through the whole wavelength range.

**Figure 2:**
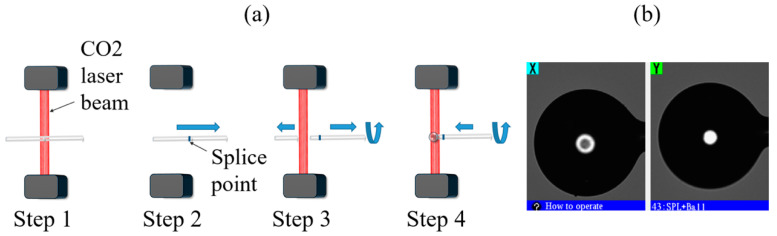
Ball resonator fabrication process. (**a**) Schematics of the fabrication process using a CO_2_ laser. (**b**) Photographic image of one resonator from X and Y cameras.

In recent years, the CO_2_ laser as a heat source has become widely used for fiber splicing and optical fiber fabrication of various geometries for several applications. Compared to other techniques, in the above-mentioned method, the outer layer of the glass absorbs the power from the CO_2_ laser, which allows changing the shape of the fiber. The process also enables production of devices with no debris and minor damage on the glass surface. Microsphere structure is formed by simultaneous surface tension and rotation when feeding an optical fiber into the CO_2_ laser operation area. After splicing two fibers, the single produced structure is then subjected to strong laser power, which breaks the fiber close to the splicing point, and this leads to the ball lens fabrication process, shown in Figure 2.

The exploitation of this fabrication method is advantageous thanks to its repeatability and low maintenance in the production of ball lens resonators.

The CO_2_ laser heat source parameters used in this study, such as ball geometry, adjustment, heating power, rotation speed, and feeding speed, are listed in the table below (Table 1).

With the proposed method, we fabricated several samples of ball resonators, achieving different size and ellipticity. In this work, we reported four samples that illustrated the capability of this method well, having diameters of 547, 532, 494, and 466 μm.

**Figure 3:**
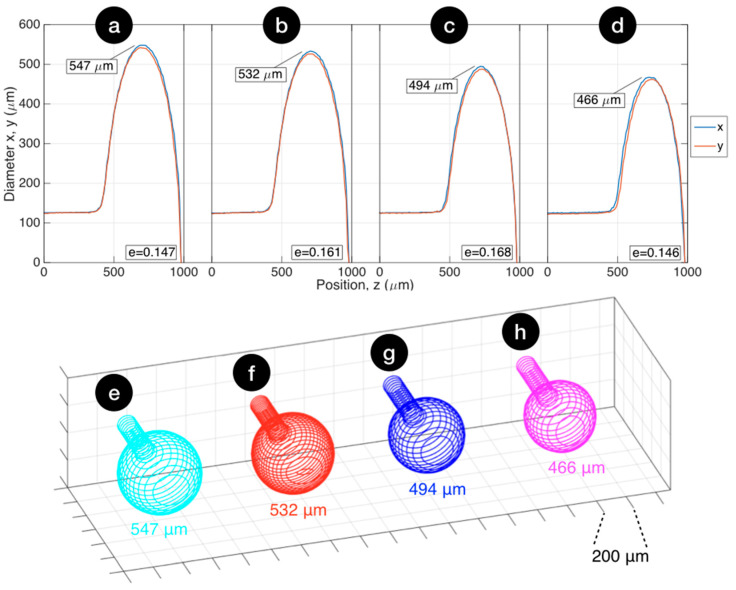
Geometrical profile of the four ball resonators reported in this work. (**a**–**d**) Ball lens profilometry reporting the diameter on the horizontal and vertical axes (x, y) for each position (z) along the fiber axis, as estimated by the fiber measurement tool of the splicer. The chart reports the maximum diameter size and the ellipticity coefficient e. (**e**,**f**) Three-dimensional reconstruction of each ball resonator, expanding the profilometry data on each z plane. Resonator diameters: (**a**,**e**) 547 μm; (**b**,**f**) 532 μm; (**c**,**g**) 494 μm; (**d**,**h**) 466 μm.

Figure 3 shows the profilometric results of ball resonator characterization performed with the measurement tool of the CO_2_ laser splicer. In the first chart, we report the estimated fiber diameter along the horizontal (x) and vertical (y) axes for each fiber position z along the input fiber axis. We observed that the method allowed fabrication of spheres with a low eccentricity error (eccentricity 0.146–0.168, corresponding to a maximum deviation of 7 μm between the x and y profiles). The transition between the input 125 μm SMF and the spherical resonator was limited to a ~40 μm region along z, and the misalignment between the ball resonator and the input fiber center was negligible (2 μm). The second chart shows a 3D reconstruction of the ball resonator, rendered with 2D profilometry on each plane parallel to z. We observed that the resonator appeared to have an almost ideal shape with a similar eccentricity error (all devices were slightly more extended along the x-direction).

As ball resonators behave as weak interferometers, with return loss (RL) ≤ −50 dB, the interrogation is based on OBR, which allows for the detection of weak spectral levels [30]. The OBR is a high-resolution optical frequency-domain reflectometer that resolves reflection spectra along the whole fiber length [31]. OBR settings used in experiments are laser scan range 1525–1610 nm, integration width 20 cm, resolution bandwidth 1.03 GHz (8.25 pm), no electrical amplification, and noise level −101 dB. In order to reduce OBR noise, spectra have been filtered with a digital low-pass filter (Butterworth 5th order, cut-off 0.005); spectral analysis was carried out in the 1540–1560 m range to eliminate the transients of the filter.

**Figure 4:**
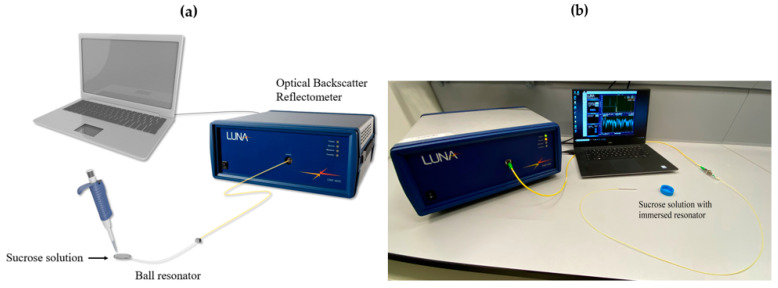
Refractive index measurement with sucrose solution. (**a**) Schematics of measurement procedure setup. (**b**) Photographic image measurement procedure setup.

The resonators were calibrated for refractive index change with a sucrose solution. Every microresonator was placed in a 10% sucrose solution and change was observed. This was followed by the addition of 100 μL of 40% sucrose to the 10% sucrose solution (total nine increments), with RI values increasing from 1.34783 to 1.34922 [11], as shown in Figure 4.

Sensitivity to RI was measured both in wavelength and amplitude domains, measuring the wavelength shift and the change of intensity through a centroid algorithm, as described in [32].

## 3. Results

**Figure 5:**
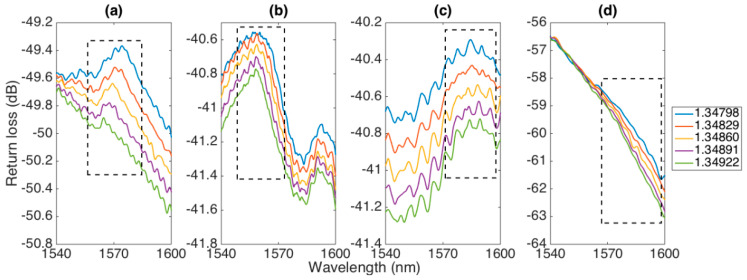
Reflection spectra of the different ball resonators, reporting return loss in the 1540–1600 nm range for different RI values (1.34798–1.34922). The window in each chart shows the spectral feature interrogated by the system. Ball resonator diameters: (**a**) 547 μm; (**b**) 532 μm; (**c**) 494 μm; (**d**) 466 μm.

The spectra of the four ball resonators described in the previous section, as a function of RI change, are shown in Figure 5. At first, we could evaluate the spectral shape and the reflectivity levels of the spectra. The 547 μm ball resonator had a return loss of −49.4 dB, and the spectrum appeared with a shallow reflection peak around 1570 nm, with amplitude and wavelength that changed as a function of the RI. The fringe visibility of the peak was shallow, around 0.2 dB for the lowest RI. The change of amplitude occurring with RI change was more noticeable, as the spectral level of the inner peak decreased with the RI increase. The 532 μm ball resonator showed two peaks within the interrogation range, around 1558 and 1592 nm, with similar fringe visibility of 0.2 dB; the reflectivity was higher than the first resonator (RL −40.6 dB in the peak). The third resonator with 494 μm diameter had a different spectral envelope, characterized by the presence of several fluctuations in the spectrum. The spectral shape showed a peak around 1574 nm, which had a 0.3 dB fringe over the background level. Finally, the last resonator presented in this work (466 μm) had a spectrum different from the previous samples, which appeared as a linear shape, being the part of a larger fringe. The return loss was about −56.5 dB in the highest level in the window and dropped to −63 dB at the longest wavelength.

Overall, spectral analysis suggested elements of randomness, implicit in the weak interferometric principle, occurring within the ball resonator. Low reflectivity value at the interface between the fiber compound and the outer medium (~0.14%), as well as imperfections in the spherical shape, highlighted in Figure 3, contributed to generate reflection spectra that had a different envelope in each spectrum, almost resembling a random signal. Compared to other low-finesse interferometers characterized by broad bandwidth and a periodic spectrum [33], ball resonator spectra have shallow fringes and a less predictable spectrum with different spectral features for each sample.

Nevertheless, interrogation of ball resonators can be performed with a method similar to other interferometers, i.e., by either tracking the peak (or dip) wavelength of a specific spectral feature or by measuring the change of spectral intensity [34]. We reported both methods in the following text, applied to the features highlighted in Figure 5: in the first three sensors, the main spectral peak was tracked, while in the fourth sensor, in the absence of a clear peak in the window of the interrogator, the slope on the longer wavelength side was detected.

**Figure 6:**
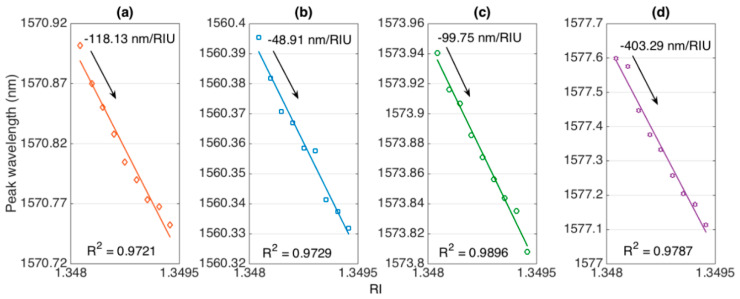
Evaluation of wavelength shift sensitivity for each ball resonator. The charts show the estimated peak wavelength for each interrogated spectral feature for different RI values (1.34798–1.34922). The sensitivity was evaluated as the slope of linear regression, R^2^ = coefficient of determination. Ball resonator diameters: (**a**) 547 μm; (**b**) 532 μm; (**c**) 494 μm; (**d**) 466 μm.

Figure 6 shows wavelength sensitivity for each sensor, reporting the peak wavelength estimated with the centroid algorithm for different RI values (on a 1.24 × 10^−3^ interval). For all sensors, we observed a linear response, where peak wavelength decreased with RI increase. The sensitivity was measured with linear regression, reporting the coefficient of determination R^2^. For all sensors, a very linear trend was observed, with R^2^ > 0.97. Sensitivity values were estimated as −118.13 nm/RIU (547 μm), −48.91 nm/RIU (532 μm), −99.75 nm/RIU (494 μm), and −403.29 nm/RIU (466 μm); the last value appeared higher, partly due to the different shape of the interrogated spectral feature.

**Figure 7:**
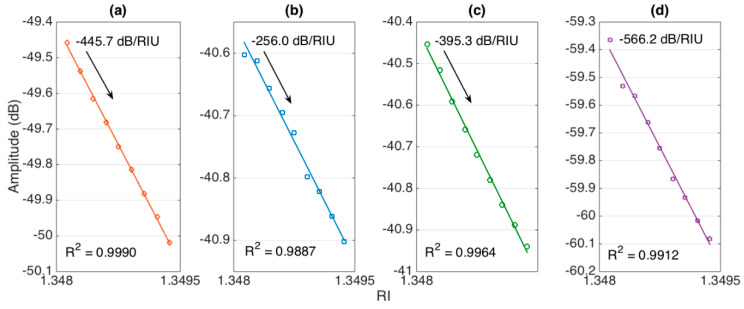
Evaluation of amplitude-level sensitivity for each ball resonator. The charts show the spectral amplitude of the spectral features for different RI values (1.34798–1.34922). The sensitivity was evaluated as the slope of linear regression, R^2^ = coefficient of determination. Ball resonator diameters: (**a**) 547 μm; (**b**) 532 μm; (**c**) 494 μm; (**d**) 466 μm.

Similarly, Figure 7 shows amplitude sensitivity estimated for each sensor on the same RI interval. Again, we observed a linear trend (R^2^ about 0.99 for all sensors), with intensity reducing as a function of RI for all interrogated spectral features. The estimated sensitivity figures were −445.7 dB/RIU (547 μm), −256.0 nm/RIU (532 μm), −395.3 nm/RIU (494 μm), and −566.2 nm/RIU (466 μm), respectively.

Table 2 reviews the parameters of all four sensors included in this analysis. Quantitatively, the sensitivity figures obtained with this method were similar to other interferometers, despite the randomness of the spectra and shallow spectral envelopes. Low values of reflectivity required the use of an interrogator capable of resolving low power levels, such as the OBR, for this analysis.

## 4. Conclusions

In conclusion, we reported the fabrication and interrogation of fiber-optic ball resonator structures designed for RI detection. The sensing unit was based on a spherical structure of ~500 μm diameter fabricated on the tip of an SMF with a CO_2_ laser splicer through a rapid, robust, and repeatable process. The obtained spectra were different for each sensor, as each resonator had a distinctive shape and spectral features; however, the interrogation could be performed by tracking the wavelength shift or the amplitude change of a spectral feature, as in standard interferometers.

We reported four different samples to show the fabrication process and highlight the spectral characteristics. The reported sensors (466–547 μm diameter) had sensitivity of 48.9–403.3 nm/RIU and 256.0–566.2 dB/RIU. The sensitivity appeared to not be a direct consequence of ball resonator size, but rather it depended on the shape of the interrogated spectral features.

The ball resonators appeared as an attractive alternative to other in-fiber interferometers in terms of ease, rapidity, repeatability of fabrication, and robustness of the device in use. The fabrication of the prototypes presented in this work was a repeatable process with ~600 s, but can be easily scaled down by a factor of 10 by acting on fiber pulling speed. Compared to tapers or etched fibers, the mechanical structure of the sensor was robust. Overall, ball resonators interrogated via OBR are promising structures for optical fiber biosensors, as the spherical surface is suitable to be functionalized through thin-film metallic layer deposition. Future work will explore the selective application of ball resonators in biosensors and optimization of the interrogation to automatically track the monitored spectral features.

## Figures and Tables

**Table 1:** Parameters used for fabrication of ball resonators with diameters of 547, 532, 494, and 466 μm.

Fabrication Parameter	547 μm	532 μm	494 μm	466 μm
Diameter adjustment	−10	20	−10	20
Pre-Heat	1	1	1	0
Absolute Power	368	372	368	388
Relative Power	184	250	290	300
Break Add Power	400	450	368	455
Feeding Speed	0.02	0.02	0.02	0.05
Rotator Speed	150	150	150	150

**Table 2:** Figures of merit and performance metrics of the four ball resonators reported in this work.

No.	Diameter (μm)	Max. RL (dB)	Wavelength Shift		Amplitude Change	
			Sensitivity (nm/RIU)	R^2^	Sensitivity (dB/RIU)	R^2^
1	547	−49.4	−118.13	0.9872	−445.7	0.9990
2	532	−40.6	−48.91	0.9729	−256.0	0.9887
3	494	−40.4	−99.75	0.9896	−395.3	0.9964
4	466	−56.6	−403.29	0.9787	−566.2	0.9912
